# Method for the Manual Analysis of Moiré Structures in STM images

**DOI:** 10.1002/cphc.202001034

**Published:** 2021-05-04

**Authors:** Sebastian Günther, Patrick Zeller, Bernhard Böller, Joost Wintterlin

**Affiliations:** ^1^ Fakultät für Chemie Technische Universität München Lichtenbergstr. 4 85748 Garching Germany; ^2^ Elettra – Sincrotrone Trieste S.C.p.A. SS14 – km 163.5 34149 Basovizza Trieste Italy; ^3^ Department Chemie Ludwig-Maximilians-Universität München Butenandtstr. 5–13 81377 Munich Germany; ^4^ Center for NanoScience Schellingstr. 4 80799 Munich Germany; ^5^ Current address: Helmholtz-Zentrum Berlin für Materialien und Energie GmbH, BESSY II Albert-Einstein-Straße 15 12489 Berlin Germany; ^6^ Fritz-Haber-Institut der Max-Planck-Gesellschaft, Dept. Inorganic Chemistry Faradayweg 4–6 14195 Berlin Germany

**Keywords:** two-dimensional solids, graphene, layered materials, moiré structures, scanning tunneling microscopy

## Abstract

A method is presented to manually determine the lattice parameters of commensurate hexagonal moiré structures resolved by STM. It solves the problem that lattice parameters of moiré structures usually cannot be determined by inspection of an STM image, so that computer‐based analyses are required. The lattice vector of a commensurate moiré structure is a sum of integer multiples both of the two basis vectors of the substrate and of the adsorbed layer. The method extracts the two factors with respect to the adsorbed layer from an analysis of the Fourier transform of an STM image. These two factors are related to the two factors with respect to the substrate layer. Using the cell augmentation method, six possible moiré structures are identified by algebra. When the orientation and lattice constant of the substrate are roughly known, this information is usually sufficient to determine a unique moiré structure and its lattice parameters.

## Graphical abstract

**Manual analysis**: A method is presented to analyze STM images of commensurate, hexagonal moiré structures on surfaces. Precise lattice constants and rotational angles can usually not be derived by simple inspection. However, the equations describing moiré structures can be solved analytically, from which a step‐by‐step recipe is developed that gives exact lattice parameters. Trial‐and‐error approaches as well as numerical computations are avoided. The method makes use of the Fourier transform, as illustrated for the simulated data in the table of content image.

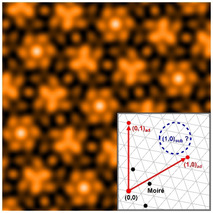

## 1 Introduction

When two 2D lattices are superimposed that slightly differ in their lattice constants and/or are rotated with respect to each other, a moiré structure results. On single crystal surfaces the effect has been known for more than 40 years[^1^, ^2^, ^3^, ^4^] and the number of reported examples has strongly grown in recent years. Moiré structures have been observed, e. g., for adsorbed close‐packed monolayers of noble gas atoms[^3^, ^5^, ^6^] and of C_60_ ^[7]^ and CO molecules.[^8^, ^9^] For epitaxially grown graphene on metal surfaces they even are a general observation.[^10^, ^11^, ^12^, ^13^, ^14^, ^15^, ^16^, ^17^, ^18^, ^19^, ^20^, ^21^] Moiré structures have also been reported for epitaxial layers of other 2D materials such as h‐BN,^[22]^ surface oxides[^2^, ^23^] and sulfides,^[24]^ and they have been found for heteroepitaxial metal films,^[25]^ misoriented graphite surfaces^[26]^ and for surface reconstructions.[^1^, ^27^]

Which moiré structure is formed in a particular case depends on the interactions within the adsorbed layer and between the adsorbed layer and the surface underneath, and there have been efforts by theory, based on considerations about the nature of the interactions, to predict which moiré structure may result.[^28^, ^29^, ^30^, ^31^, ^32^, ^33^, ^34^, ^35^]

A more basic question, which is independent of the specific interactions, is how the lattice parameters of a moiré structure depend on the symmetry and on the lattice constants of the two superimposed lattices and on their relative rotational angle, and how one can classify the structure. This question has also been addressed by theory. The analyses were mostly focused on commensurate moiré structures, i. e., on those cases where the moiré lattice vectors can be written as sums of integer multiples of the lattice vectors of the substrate and also of the adsorbed layer. For commensurate moiré structures formed by two hexagonal lattices, Tkatchenko introduced the so‐called hexagonal number sequence 1, 3, 4, 7, …^[35]^ The numbers represent the factors by which the unit cell of the adsorbed layer can be enlarged in such a way that an integer number of hexagonally arranged particles can be filled in. In this way a series of moiré structures can be constructed that can exist for hexagonal systems. Hermann presented a treatment, which was not restricted to hexagonal systems, in which he analyzed the $\left(2x2\right)$ matrix $\underline{M}$ that relates the unit vectors of the substrate lattice to the unit vectors of the moiré lattice.[^36^, ^37^] Depending on the symmetry of the system and on the ratio of the lattice constants of the adsorbed layer and the substrate layer and on the rotational angle, one can calculate the matrix elements and generate possible moiré structures. In publications by two of us, moiré structures were treated in analogy to an acoustic beat of two different frequencies, and a Fourier series representation of the two superimposed lattices was used.[^38^, ^39^] By means of the convolution theorem of Fourier transformation, explicit equations for the moiré beating frequencies were derived. The frequencies can be classified in acoustic terms; first order moiré structures result from the beating of fundamental tones, and higher‐order structures from the beating of overtones. Artaud et al. demonstrated that the unit cell of a moiré structure may contain more than one beating.^[40]^ Later, it was shown that such cases can be treated by the so‐called cell augmentation method, by which the difficult handling of higher‐order structures is replaced by a first‐order treatment.^[39]^

In principle, all of these theoretical works dealt with the question which moiré structures result when one varies the ratios of the lattice constants of the two superimposed lattices and the relative rotational angles. By contrast, in an experiment, where one deals with a given moiré structure, one has to solve, in a sense, a reverse problem. We here consider experiments by scanning tunneling microscopy (STM) that has provided most of the data. An STM image of a moiré structure typically shows an atomic fine structure from the adsorbed layer that is modulated by a long‐wave pattern from the moiré structure, and one has to find out which lattice constant and rotational angle of the adsorbed layer with respect to the substrate can generate the observed moiré pattern. If successful, such an analysis can give very precise lattice parameters of the adsorbed layer, a result of the property of moiré structures to amplify differences between the two involved lattices.

However, the direct analysis of a moiré pattern in an STM image, simply by counting the number of atomic features in a unit cell of the moiré pattern and measuring the angles, is problematic. The quality of STM data, even when there are only moderate interferences from noise, tip changes, thermal drift, and piezo creep, is usually not sufficient for discriminating between the many different moiré structures possible. From a direct analysis it is also difficult to say whether the moiré modulation seen in an STM image actually reflects a translationally symmetric structure or whether it is only apparently translationally symmetric, and the actual lattice is a multiple of it or even incommensurate. Moreover, there is the fundamental problem that the STM does not “see” the lattice of the substrate underneath the adsorbed layer. Separate reference experiments have to be performed, causing an uncertainty in the relative orientations of the two lattices. The errors depend on the experiment, but even when they are low, a unique set of exact lattice parameters of the moiré structure with respect to the substrate can often not be extracted. Some of these problems can be resolved when the Fourier transform is used instead of the real‐space image.^[40]^ In particular, the precision is enhanced by an averaging effect of the Fourier transformation. However, the problem with the uncertainty about the substrate lattice persists.

More sophisticated methods for analyzing moiré structures in STM data are therefore required. One solution has been provided by the so‐called commensurability plots.[^38^, ^39^] Such plots are 3D functions on the x /φ plane (x is the ratio of the lattice constants of the two lattices and φ is the rotational angle), which are a measure of the deviations of the solutions from commensurability. At the zeros of the plots commensurate moiré structures exist. There is an infinite number of zeros, but if one can constrain, using the experimental data, the range of x and φ values, a unique solution can often be found. However, one has to draw a complicated 3D function, and for higher‐order moiré structures the equations can only be solved numerically. Recently, Le Ster et al. have made a software package available, also based on the convolution theorem of Fourier transformation, by which one can analyze STM images of graphene overlayers on arbitrary supports.^[41]^ (The program does not discriminate between commensurate and incommensurate structures.) In any case, calculations on a computer are required.

Here, we present a method for solving commensurate, hexagonal moiré structures in STM images that is much simpler. It only requires a Fourier transformation, a standard tool in STM image processing software, and a pocket calculator. We demonstrate its application for moiré structures of CO molecules on a Co(0001) surface that previously have been analyzed by means of the commensurability plot method. We also investigate the limits of the method, and for a graphene structure on Ir(111) we show that some accuracy is required to avoid wrong conclusions.

## 2 Results and Discussion

### 2.1 Setup of the Required Equations

**Figure 1:**
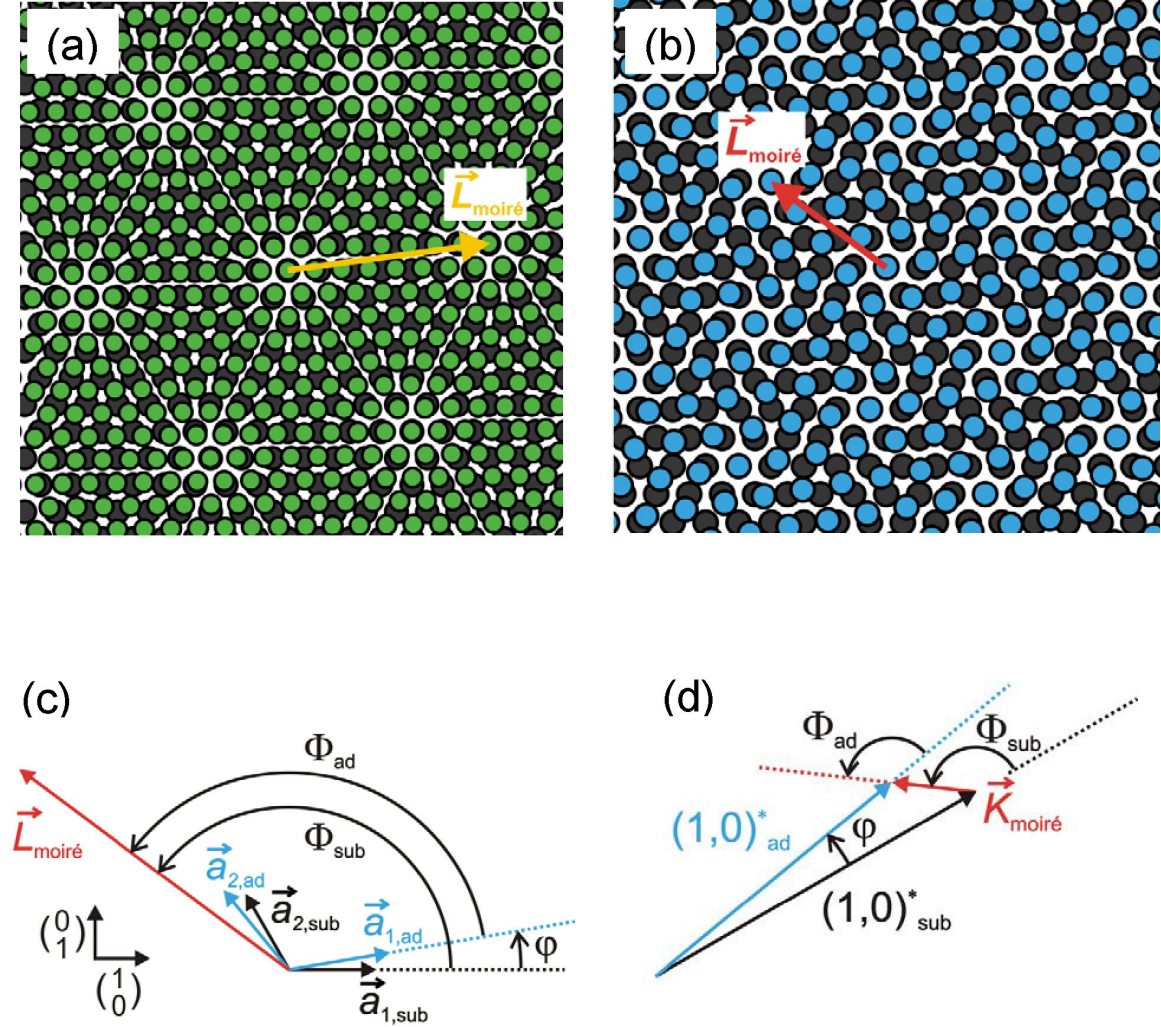
Commensurate moiré structures formed by two hexagonal lattices. a) Structure with m =7, n =1, r =8, s =1, corresponding to a “simple” cell and a shorter lattice constant of the adsorbed layer (green) than of the substrate (dark grey) (x >1). b) Structure with m =−2, n =3, r =−1, s =3, also a simple cell, but with a longer lattice constant of the adsorbed layer (blue) than of the substrate (dark grey) (x <1). c) Lattice vectors and angles of the structure of Figure 1b. ${\vec{L}}_{moir\overset{´}{e}}$ is a moiré lattice vector, ${\vec{a}}_{1,\mathrm{sub}}$ and ${\vec{a}}_{2,\mathrm{sub}}$ are the basis vectors of the substrate, and ${\vec{a}}_{1,\mathrm{ad}}$ and ${\vec{a}}_{2,\mathrm{ad}}$ are the basis vectors of the adsorbed layer. d) Reciprocal lattice vectors of the structure in Figure 1b. ${\vec{K}}_{moir\overset{´}{e}}$ is the reciprocal lattice vector of the moiré structure, and ${\left(1,0\right){}^{*}}_{\mathrm{sub}}$ and ${\left(1,0\right){}^{*}}_{\mathrm{ad}}$ are the reciprocal basis vectors of the substrate and adsorbed layer, respectively. The vectors are rotated by 30° with respect to the corresponding real‐space vectors of Figure 1c, but the relative angles φ , $\Phi_{\mathrm{sub}}$ , and $\Phi_{\mathrm{ad}}$ are unchanged.

A moiré structure – examples are shown in Figures 1a and 1b – is defined by a lattice vector ${\vec{L}}_{moir\overset{´}{e}}$ that can be written as a sum of multiples of the basis vectors ${\vec{a}}_{1,sub}$ and ${\vec{a}}_{2,\mathrm{sub}}$ of the substrate

(1) $${\vec{L}}_{moir\overset{´}{e}}=m{\vec{a}}_{1,\mathrm{sub}}+n{\vec{a}}_{2,\mathrm{sub}},$$

and at the same time as a sum of multiples of the basis vectors ${\vec{a}}_{1,\mathrm{ad}}$ and ${\vec{a}}_{2,\mathrm{ad}}$ of the adsorbed layer

(2) $${\vec{L}}_{moir\overset{´}{e}}=r{\vec{a}}_{1,\mathrm{ad}}+s{\vec{a}}_{2,\mathrm{ad}}.$$

In order that the moiré structure is commensurate all four factors, m , n , r , and s , in eqs. (1) and (2) have to be integers. We here only treat the superposition of hexagonal lattices, so that the resulting moiré structure is also hexagonal. The two basis vectors ${\vec{a}}_{1}$ and ${\vec{a}}_{2}$ , both of the substrate and of the adsorbed layer, we define such that they include angles of 120° (Figure 1c). Figure 1c also shows the angle φ by which the lattice of the adsorbed layer is rotated with respect to the substrate, and the angles $\Phi_{\mathrm{sub}}$ and $\Phi_{\mathrm{ad}}$ by which the moiré lattice is rotated with respect to the lattices of the substrate and adsorbed layer, respectively. In the following we write eqs. (1) and (2) in the short forms

(1′) $${\vec{L}}_{moir\overset{´}{e}}=\left(m,n\right),$$

(2′) $${\vec{L}}_{moir\overset{´}{e}}=\left(r,s\right).$$

The problem we want to solve here is to obtain the integer factorsm , n , r , and s from an analysis of a moiré structure in an STM image. When these factors have been identified all lattice parameters of the commensurate moiré structure can be determined.

Most of the required equations have already been derived in two previous publications.[^38^, ^39^] By using the cartesian coordinate system indicated in Figure 1c, the substrate basis vectors can be written as ${\vec{a}}_{1,\mathrm{sub}}=a_{\mathrm{sub}}\left(\begin{array}{c} 1\\ 0 \end{array}\right)$ and ${\vec{a}}_{2,\mathrm{sub}}=a_{\mathrm{sub}}\left(\begin{array}{c} -1/2\\ \sqrt{3}/2 \end{array}\right).\;$ Insertion into eq. (1) gives

(1′′) $${\vec{L}}_{moir\overset{´}{e}}=a_{\mathrm{sub}}\left(\begin{array}{c} m-n/2\\ n\sqrt{3}/2 \end{array}\right).$$

${\vec{L}}_{moir\overset{´}{e}}$ can also be expressed by its length $L_{\mathrm{moir}\overset{´}{e}}$ and rotational angle $\Phi_{\mathrm{sub}}$ , ${\vec{L}}_{moir\overset{´}{e}}=L_{\mathrm{moir}\overset{´}{e}}\left(\begin{array}{c} \cos\left(\Phi_{\mathrm{sub}}\right)\\ \sin\left(\Phi_{\mathrm{sub}}\right) \end{array}\right)=l\;a_{\mathrm{sub}}\left(\begin{array}{c} \cos\left(\Phi_{\mathrm{sub}}\right)\\ \sin\left(\Phi_{\mathrm{sub}}\right) \end{array}\right)$ , where l is the length of ${\vec{L}}_{moir\overset{´}{e}}$ in units of the substrate lattice constant $a_{\mathrm{sub}}$ . Equating this expression with ${\vec{L}}_{moir\overset{´}{e}}$ from eq. (1′′) leads to two equations, which, solved for m and n , give

(3) $$m=\;\frac{1}{\sqrt{3}}l\sin\left(\Phi_{\mathrm{sub}}\right)+l\cos\left(\Phi_{\mathrm{sub}}\right),$$

(4) $$n=\frac{2}{\sqrt{3}}l\sin\left(\Phi_{\mathrm{sub}}\right).$$

The equations for r and s are obtained equivalently, taking into account that the coordinate system has to be rotated by φ . The results are

(5) $$r=\;\frac{1}{\sqrt{3}}lx\sin\left(\Phi_{\mathrm{sub}}-\varphi \right)+lx\cos\left(\Phi_{\mathrm{sub}}-\varphi \right),$$

(6) $$s=\frac{2}{\sqrt{3}}lx\sin\left(\Phi_{\mathrm{sub}}-\varphi \right).$$

The equations contain the additional parameter x , defined as the ratio of the lattice constants of the substrate and the adsorbed layer, $x=a_{\mathrm{sub}}/a_{\mathrm{ad}}$ .

It has also been shown that a first order moiré structure, which results from a convolution of two Fourier expansions that only contain first order coefficients, fulfills the condition^[38]^

(7) $${\vec{K}}_{moir\overset{´}{e}}={{\left(1,0\right)}^{{}^{*}}}_{ad}-{{\left(1,0\right)}^{{}^{*}}}_{sub}.$$

Equation (7) defines a triangle in reciprocal space (Figure 1d) that is formed by ${\vec{K}}_{moir\overset{´}{e}}$ , a reciprocal lattice vector of the moiré structure, by ${\left(1,0\right){}^{*}}_{ad}$ , a reciprocal basis vector of the adsorbed layer, and by ${\left(1,0\right){}^{*}}_{sub}$ , a reciprocal basis vector of the substrate. Applying the cosine theorem to this triangle leads to two equations that relate l and $\Phi_{\mathrm{sub}}$ to φ and x :

(8) $$l=\frac{1}{\sqrt{1+x^{2}-2x\cos\left(\varphi \right)}},$$

and

$$\Phi_{\mathrm{sub}}=\arccos\left[\frac{x\cos\left(\varphi \right)-1}{\sqrt{1+x^{2}-2x\cos\left(\varphi \right)}}\right]\;\;\mathrm{for}\;\varphi \geq 0^{\circ },$$

(9) $$\Phi_{\mathrm{sub}}=-\arccos\left[\frac{x\cos\left(\varphi \right)-1}{\sqrt{1+x^{2}-2x\cos\left(\varphi \right)}}\right]\;\mathrm{for}\;\varphi <0^{\circ }.$$

The equations are valid for x>1 (Figure 1a) as well as for x=1 and x<1 (Figure 1b), all three of which cases occur in physical systems. For example, epitaxially grown graphene on transition metals mostly has a shorter lattice constant than the substrate (x>1 ), whereas the case x =1 is realized by twisted bilayer graphene, and an example for x<1 is a close‐packed layer of CO molecules on Co(0001). In eq. (9) a case distinction is made, according to which $\Phi_{\mathrm{sub}}$ changes sign for a positive or negative φ . We here define $\varphi \geq 0^{\circ }$ as an anti‐clockwise rotation [Figures 1a and 1b], and $\varphi <0^{\circ }$ as a clockwise rotation. Inserting eqs. (8) and (9) into eqs. (3) to (6) gives four functions $m\left(x,\varphi \right)$ , $n\left(x,\varphi \right)$ , $r\left(x,\varphi \right)$ , and $s\left(x,\varphi \right)$ that in previous analyses have been used to generate the so‐called first‐order commensurability plots.[^38^, ^39^]

Here we instead make use of the fact that the factors m and n from the substrate lattice are related in a systematic way to the factors r and s from the lattice of the adsorbed layer. The relations follow from an analysis of eqs. (3) to (6). For this purpose, first the case $\varphi \geq 0^{\circ }$ is considered and addition theorems are applied to the sine and cosine terms containing $\left(\Phi_{\mathrm{sub}}-\varphi \right)$ in eqs. (5) and (6). Then all $sin\left(\Phi_{\mathrm{sub}}\right)$ terms are replaced by $\sin\left(\Phi_{\mathrm{sub}}\right)=\;\sqrt{1-{cos}^{2}\left(\Phi_{\mathrm{sub}}\right)}$ , and l is inserted from eq. (8) and $\cos\left(\Phi_{\mathrm{sub}}\right)$ from eq. (9). The results are

(10) $$m=\;\frac{1}{\sqrt{3}}l^{2}x\sin\left(\varphi \right)+l^{2}x\cos\left(\varphi \right)-l^{2},$$

(11) $$n=\;\frac{2}{\sqrt{3}}l^{2}x\sin\left(\varphi \right),$$

(12) $$r=\;\frac{1}{\sqrt{3}}l^{2}x\sin\left(\varphi \right)+l^{2}x^{2}-l^{2}x\cos\left(\varphi \right),$$

(13) $$s=\;\frac{2}{\sqrt{3}}l^{2}x\sin\left(\varphi \right).$$

By squaring eq. (8) one can write $1=l^{2}\left[1+x^{2}-2x\cos\left(\varphi \right)\right]$ and subtract this expression from r

(14) $$r-1=\;\frac{1}{\sqrt{3}}l^{2}x\sin\left(\varphi \right)+l^{2}x\cos\left(\varphi \right)-l^{2}.$$

When one then compares eq. (10) with eq. (14) and eq. (11) with eq. (13) one finds that the results are identical, i. e.

(15) m=r-1,

(16) n=s.

For the case $\varphi <0^{\circ }$ some terms in eqs. (10) to (14) change signs, but the results, eqs. (15) and (16), are identical. These relations between m , n , r , and s apply to simple cells, i. e., to cases where the long‐wave moiré modulation seen in an STM image reflects a translationally symmetric structure. However, it is often observed that the long‐wave modulation does not reflect an actual translationally symmetric structure. This does not necessarily mean an incommensurate structure. More frequent is the case that the moiré structure is still commensurate but that the actual unit cell is larger than the simple cell by an integer multiple.[^40^, ^42^, ^43^] In hexagonal systems the possible enhancement factors are given by the hexagonal number sequence 1, 3, 4, 7, …^[35]^ The lattice vector of a tripled structure is $\sqrt{3}$ times longer than of a simple cell and rotated by 30°, and for the case of a 4 times larger cell it is by a factor of 2 longer and aligned.

For these larger cells the relations between m , n , r , and s change.^[39]^ One can see this, for the tripled structure, by constructing the $\sqrt{3}$ times longer and 30° rotated moiré lattice vector by adding two vectors of the simple periodicity, namely $\left(m,n\right)$ and the 60° rotated vector $\left(m-n,m\right)\;$ (for the derivation of the rotated vectors see below, Table 2):

(17) $${\vec{L}}_{moir\overset{´}{e}}=\left(m,n\right)+\left(m-n,m\right)=\left(2m-n,m+n\right)=\left(\tilde{m},\tilde{n}\right),$$

which leads to two new factors $\tilde{m}$ and $\tilde{n}$ . Correspondingly, for the moiré vector expressed in the unit vectors of the adsorbate layer, one obtains two new factors $\tilde{r}$ and $\tilde{s}$

(18) $${\vec{L}}_{moir\overset{´}{e}}=\left(r,s\right)+\left(r-s,r\right)=\left(2r-s,r+s\right)=\left(\tilde{r},\tilde{s}\right).$$

By inserting m=r-1 [eq. (15)] and n=s [eq. (16)] into eq. (17) and using the results for $\tilde{r}$ and $\tilde{s}$ from eq. (18) one obtains the relations between these new multiplication factors:

(19) $$\tilde{m}=2m-n=2\left(r-1\right)-s=\left(2r-s\right)-2=\tilde{r}-2,$$

(20) $$\tilde{n}=m+n=\left(r-1\right)+s=\left(r+s\right)-1=\tilde{s}-1.$$

For the quadrupled cell the moiré vector of the simple periodicity $\left(m,n\right)$ has to be doubled:

(21) $${\vec{L}}_{moir\overset{´}{e}}=2\left(m,n\right)=\left(2m,2n\right)=\left(\tilde{m},\tilde{n}\right),$$

(22) $${\vec{L}}_{moir\overset{´}{e}}=2\left(r,s\right)=\left(2r,2s\right)=\left(\tilde{r},\tilde{s}\right),$$

which, with eqs. (15) and (16), gives

(23) $$\tilde{m}=2m=2\left(r-1\right)=2r-2=\tilde{r}-2,$$

(24) $$\tilde{n}=2n=2s=\tilde{s}.$$

Larger cells may be treated correspondingly. Eqs. (15), (16), (19), (20), (23), and (24) are collected in Table 1 where we have renamed the factors $\tilde{m}$ , $\tilde{n},\;\tilde{r}$ , and $\tilde{s}$ to factors without tilde. The table will be used below for converting ${\vec{L}}_{moir\overset{´}{e}}=\left(r,s\right)$ into ${\vec{L}}_{moir\overset{´}{e}}=\left(m,n\right)$ .

**Table 1:** Moiré lattice vectors for the simple cell and for the cells enlarged by factors of 3 and 4.

Augmentation factor	${\vec{L}}_{moir\overset{´}{e}}$ expressed in the unit vectors of the adsorbed layer	${\vec{L}}_{moir\overset{´}{e}}$ expressed in the unit vectors of the substrate
1	(r,s)	$\left(m,n\right)=\left(r-1,s\right)$
3	(r,s)	$\left(m,n\right)=\left(r-2,s-1\right)$
4	(r,s)	$\left(m,n\right)=\left(r-2,s\right)$

For the analysis we furthermore need moiré lattice vectors that are rotated by multiples of 60° with respect to ${\vec{L}}_{moir\overset{´}{e}}$ . These vectors are obtained by applying a rotation matrix with angles of 60°, 120°, etc. to ${\vec{L}}_{moir\overset{´}{e}}$ . The results, both expressed in unit vectors of the adsorbed layer lattice and of the substrate lattice vectors, are listed in Table 2.

**Table 2:** Rotated moiré vectors obtained by applying a rotation matrix to ${\vec{L}}_{moir\overset{´}{e}}$ with the indicated angles.

Rotation angle	${\vec{L}}_{moir\overset{´}{e}}$ expressed in the unit vectors of the adsorbed layer	${\vec{L}}_{moir\overset{´}{e}}$ expressed in the unit vectors of the substrate
0°	(r,s)	$\left(m,n\right)$
60°	$\left(r-s,r\right)$	$\left(m-n,m\right)$
120°	$\left(-s,r-s\right)$	$\left(-n,m-n\right)$
180°	$\left(-r,-s\right)$	$\left(-m,-n\right)$
240°	$\left(-r+s,-r\right)$	$\left(-m+n,-m\right)$
300°	$\left(s,-r+s\right)$	$\left(n,-m+n\right)$

The basic idea of the method presented here is to extract r and s from the Fourier transform of an STM image, and then to evaluate m and n by means of Table 1. When the factors m , n , r , and s have been determined in this way, the full set of lattice parameters of the moiré structure can be constructed. These parameters include the length of ${\vec{L}}_{moir\overset{´}{e}}$ in units of the lattice constants of the substrate and of the adsorbed layer:

(25) $$L_{\mathrm{moir}\overset{´}{e}}/a_{\mathrm{sub}}=l=\sqrt{m^{2}+n^{2}-mn},$$

(26) $$L_{\mathrm{moir}\overset{´}{e}}/a_{\mathrm{ad}}=lx=\sqrt{r^{2}+s^{2}-rs}.$$

These two equations can be derived by applying basic vector algebra to ${\vec{L}}_{moir\overset{´}{e}}$ .^[38]^ The parameter $x=a_{\mathrm{sub}}/a_{\mathrm{ad}}$ , the ratio of the lattice constants of the substrate and the adsorbed layer, can be evaluated with the results of eqs. (25) and 26

(27) $$x=\frac{L_{\mathrm{moir}\overset{´}{e}}/a_{\mathrm{ad}}}{L_{\mathrm{moir}\overset{´}{e}}/a_{\mathrm{sub}}}=\frac{\sqrt{r^{2}+s^{2}-rs}}{\sqrt{m^{2}+n^{2}-mn}}.$$

The square of x gives the coverage Θ , defined as the number of unit cells of the lattice of the adsorbed layer with respect to the unit cells of the substrate lattice:

(28) $$\Theta =x^{2}.$$

A further lattice parameter is the angle $\Phi_{\mathrm{sub}}$ by which the moiré structure is rotated with respect to the substrate lattice (Figure 1c):

$$\Phi_{\mathrm{sub}}=\arccos\left(\frac{m-\frac{n}{2}}{\sqrt{m^{2}+n^{2}-mn}}\right)\;\mathrm{for}\;n>0,$$

$$\Phi_{\mathrm{sub}}=-\arccos\left(\frac{m-\frac{n}{2}}{\sqrt{m^{2}+n^{2}-mn}}\right)\;\mathrm{for}\;n<0,$$

$$\Phi_{\mathrm{sub}}=0\;\mathrm{for}\;n=0\;\mathrm{and}\;m>0,$$

(29) $$\Phi_{\mathrm{sub}}=-180^{\circ }\;\mathrm{for}\;n=0\;\mathrm{and}\;m<0.$$

The equations have also been derived previously (except for the case discrimination).^[39]^ The corresponding result for $\Phi_{\mathrm{ad}}$ , the rotational angle between the moiré lattice and the lattice of the adsorbed layer (Figure 1c), is

$$\Phi_{\mathrm{ad}}=\arccos\left(\frac{r-\frac{s}{2}}{\sqrt{r^{2}+s^{2}-rs}}\right)\;\mathrm{for}\;s>0,$$

$$\Phi_{\mathrm{ad}}=-\arccos\left(\frac{r-\frac{s}{2}}{\sqrt{r^{2}+s^{2}-rs}}\right)\;\mathrm{for}\;s<0,$$

$$\Phi_{\mathrm{ad}}=0\;\mathrm{for}\;s=0\;\mathrm{and}\;r>0,$$

(30) $$\Phi_{\mathrm{sub}}=-180^{\circ }\;\mathrm{for}\;s=0\;\mathrm{and}\;r<0.$$

Finally, the angle φ by which the adsorbed layer is rotated with respect to the substrate lattice is obtained by taking the difference of $\Phi_{\mathrm{sub}}$ and $\Phi_{\mathrm{ad}}$ (Figure 1c)

(31) $$\varphi =\Phi_{\mathrm{sub}}-\Phi_{\mathrm{ad}}.$$

### 2.2 Recipe for the Application of the Method and Examples

By applying the above equations and working with the Fourier transforms we derive a recipe by which one can manually analyze commensurate hexagonal moiré structures in STM data. We demonstrate its application by means of four examples.

The first two examples are from experiments on Co(0001) which were performed in CO atmospheres at pressures between 100 and 800 mbar and at a sample temperature of 300 K.^[9]^ Under these conditions the adsorbed CO molecules form hexagonal, close‐packed monolayers on the cobalt surface. The van‐der‐Waals diameter of CO is larger than the lattice constant of the Co(0001) surface of 2.507 Å, which leads to a mismatch of the CO lattice with the lattice of the Co(0001) surface. In addition, the CO layer is rotated with respect to the substrate, giving rise to several moiré structures.

#### 2.2.1 Moiré Structure of CO Molecules on Co(0001) Formed at 800 mbar (Example 1)

**Figure 2:**
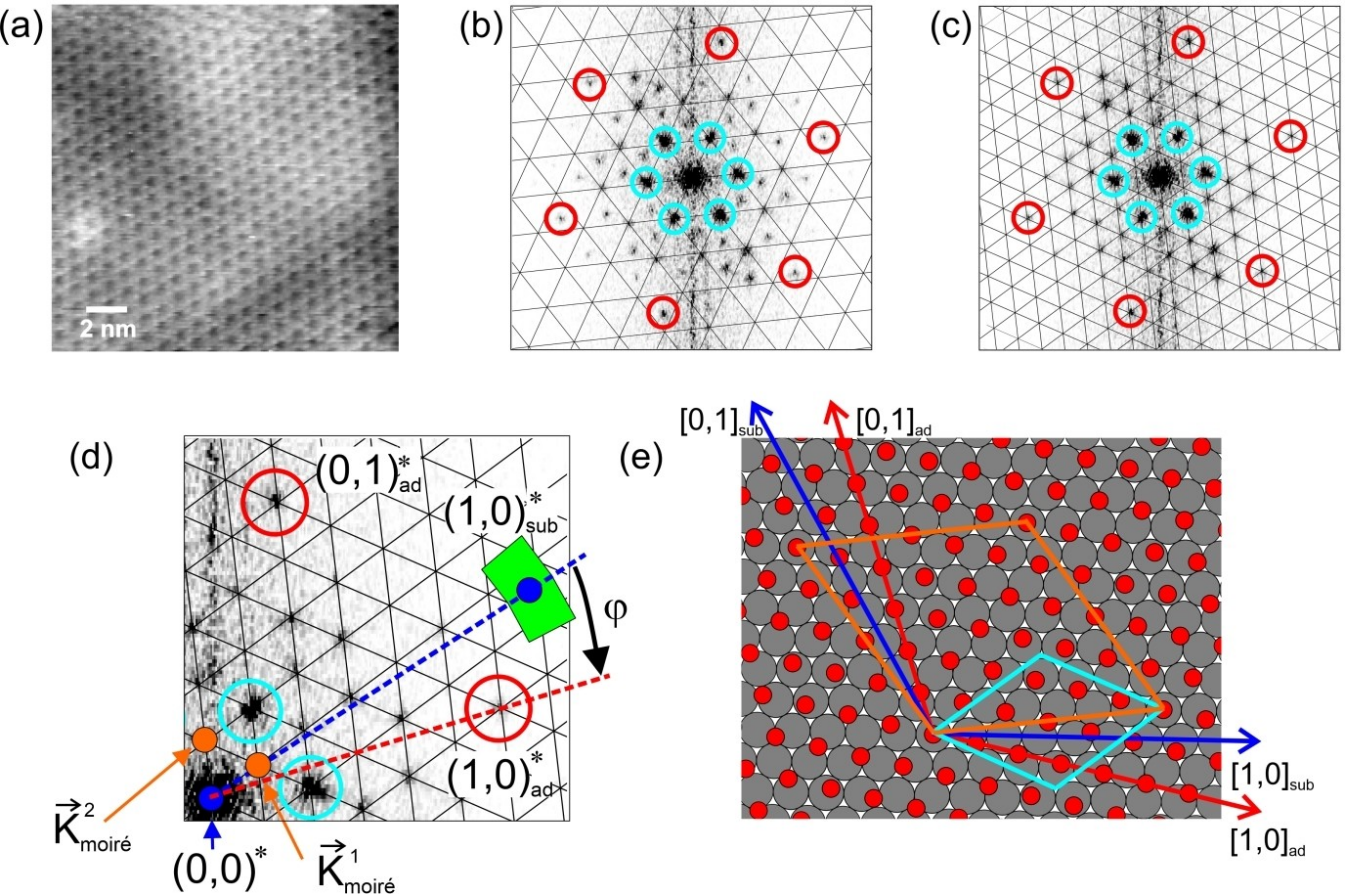
Analysis of a CO moiré structure on Co(0001) at 800 mbar CO. a) STM image, showing the long‐wave modulation from the moiré structure; *T*=300 K, tunneling tunneling voltage *V_t_*=−1.5 V, tunneling current *I_t_*=0.7 nA, image size 170 Å×170 Å. b) Fourier transform of (a). The main spots from the moiré structure are marked by blue circles, and the spots from the CO lattice are marked by red circles. The grid of fine lines is constructed from the positions of the innermost spots of the moiré structure. (Here and in the following figures, the left halves of the Fourier transforms are constructed by inversion symmetry from the right halves.) c) Same Fourier transform as (b) but with a three times finer grid. d) One quadrant of (c). ${\vec{K}}_{moir\overset{´}{e}}^{1}$ and ${\vec{K}}_{moir\overset{´}{e}}^{1}$ are the basis vectors of the reciprocal moiré lattice, and ${\left(1,0\right){}^{*}}_{ad}$ and ${\left(0,1\right){}^{*}}_{ad}$ are the basis vectors of the reciprocal CO lattice. ${\left(1,0\right){}^{*}}_{sub}$ is the approximate position of a reciprocal basis vector of the Co(0001) substrate; φ is the rotational angle of the CO lattice with respect to the substrate lattice. The green area indicates the estimated error margins. (e) Model of the $\left(\sqrt{43}\;x\;\sqrt{43}\right)R7.6^{\circ }$ structure. Red dots indicate the positions of the CO molecules; the orange diamond is a unit cell of the moiré structure, and the light blue diamond is a simple cell. (Differently from ref. [9], the Fourier transform has been corrected for some small drift such that the symmetry is exactly hexagonal.) Adapted with permission from B. Böller, P. Zeller, S. Günther, J. Wintterlin, *ACS Catal*. **2020**, *10*, 12156‐12166 (Ref. [9]). Copyright (2020) American Chemical Society.

We start with Figure 2a, an STM image recorded at 800 mbar. The hexagonal pattern is the moiré structure, and the fine structure is caused by the CO molecules. Previously, the structure has been analyzed by means of the commensurability plot method.^[9]^ Here we use it to establish the recipe. It consists of seven steps:

(1) *On the Fourier transform of the STM image draw a hexagonal grid, using the innermost spots from the moiré structure as reference points. Check whether the spots from the adsorbate lattice fall on the intersection points of this grid. If this is the case, the grid defines the reciprocal lattice of a commensurate moiré structure. The analysis depends on the quality of the Fourier transform. It may therefore be required to cut off nonlinearities at the edges of the real‐space image from piezo creep. Removing possible line noise that enhances the diffuse background in the Fourier transform may be helpful. To simplify frequency determination, it may also be appropriate to correct for STM‐typical distortions from thermal drift or small variations in the* x *and* y *calibration factors. In the present case, where hexagonal systems are treated, the Fourier transform can be corrected such that it displays precise hexagonal symmetry*.

The Fourier transform of Figure 2a is shown in Figure 2b. The six most intense spots (marked by the blue circles) close to the origin reflect the main frequencies from the moiré structure, and the six spots marked by the red circles further outside reflect the frequencies from the CO lattice. Using the innermost moiré spots as reference points the hexagonal grid of thin lines is drawn. One can see that the spots from the CO lattice are not on the intersection points of the grid, and also many of the higher‐order spots from the moiré lattice do not fall on the intersection points. The condition from step (1) is thus not fulfilled.

(2) *If this condition is not fulfilled, shrink the grid by a factor of 3, 4, 7, and so forth (a factor from the hexagonal number sequence)*,^[35]^ *until the spots from the atomic fine structure fall on the intersection points of such a smaller grid. When this has been achieved, the grid defines the reciprocal lattice of a commensurate moiré structure. This step is identical to the cell augmentation method introduced in Ref. [39]. A good hint for a tripled or quadrupled cell is when, on the grid from the simple cell, spots appear in the centers of the triangles or at the centers of the triangle edges of the grid, respectively*.

In the example, Figure 2c shows the same Fourier transform as Figure 2b, but overlaid by a 3 times smaller grid that is rotated by 30°. One can see that now the spots from the CO lattice and also all other spots pretty exactly fall on intersection points. This grid therefore represents the actual reciprocal lattice from the moiré structure. It is by a factor of 3 smaller than the lattice constructed from the most intense moiré spots, so that, in real space, the actual moiré unit cell is by a factor of 3 larger than the apparent, simple cells in the STM image.

(3) *With the reciprocal moiré lattice identified in this way, determine the factors* r *and* s *by transforming the reciprocal lattice vectors of the moiré structure into a real‐space vector* ${\vec{L}}_{moir\overset{´}{e}}=\left(r,s\right)$ *. When one* ${\vec{L}}_{\mathrm{moir}\overset{´}{e}}$ *vector has been identified create five additional vectors by rotating* ${\vec{L}}_{moir\overset{´}{e}}=\left(r,s\right)$ *by multiples of 60°, using the* (r,s) *column of* Table 2 *. Because of the hexagonal symmetry, these six vectors are equivalent descriptions of the moiré structure*.

In the example we use one quadrant of the Fourier transform (Figure 2d) to illustrate this step. The figure shows two lattice vectors from the just obtained reciprocal moiré lattice, ${\vec{K}}_{moir\overset{´}{e}}^{1}$ and ${\vec{K}}_{moir\overset{´}{e}}^{2}$ , and two reciprocal lattice vectors from the CO layer, ${\left(1,0\right){}^{*}}_{ad}$ and ${\left(0,1\right){}^{*}}_{ad}$ . The included angles of both pairs of vectors are 60° because 120° was used for the basis vectors in real space. Using the grid constructed in step (2), one can express the reciprocal CO vectors by sums of multiples of the reciprocal moiré vectors:

(32) $${{\left(1,0\right)}^{{}^{*}}}_{ad}=6\;{\vec{K}}_{moir\overset{´}{e}}^{1}-2\;{\vec{K}}_{moir\overset{´}{e}}^{2}\mathrm{and}\;{{\left(0,1\right)}^{{}^{*}}}_{ad}=2\;{\vec{K}}_{moir\overset{´}{e}}^{1}+4\;{\vec{K}}_{moir\overset{´}{e}}^{2}.$$

Solving these two equations for ${\vec{K}}_{moir\overset{´}{e}}^{1}$ and ${\vec{K}}_{moir\overset{´}{e}}^{2}$ gives

(33) $${\vec{K}}_{moir\overset{´}{e}}^{1}=\frac{2}{14}{{\left(1,0\right)}^{{}^{*}}}_{ad}+\frac{1}{14}{{\left(0,1\right)}^{{}^{*}}}_{ad}\mathrm{and}\;{\vec{K}}_{moir\overset{´}{e}}^{2}=-\frac{1}{14}{{\left(1,0\right)}^{{}^{*}}}_{ad}+\frac{3}{14}{{\left(0,1\right)}^{{}^{*}}}_{ad},$$

which can be combined to a single equation by using the matrix 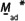

(33’)
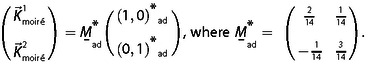



The reciprocal space matrix 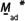 is then inverted and transposed to give the real‐space matrix 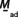 by using $m_{ij}{}^{*}$ , the matrix elements of 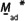, and the determinant 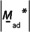:(34)
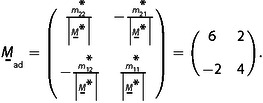



The first line of 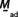 is one of the six solutions for r and s that determine the real space moiré vector with respect to the lattice vectors of the adsorbed layer, ${\vec{L}}_{\mathrm{moir}\overset{´}{e}}=\left(r,s\right)$ =(6,2). The set of six equivalent solutions is obtained by applying the 60° vector rotations of Table 2, giving ${\vec{L}}_{\mathrm{moir}\overset{´}{e}}=\left(r,s\right)$ =(6,2), (4,6), (‐2,4), (‐6,–2), (‐4,–6), and (2,–4). One could as well have chosen the second line of 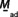 in eq. (34) as it is one of the six solutions. One could also have started, on the grid in Figure 2d, with a pair of ${\vec{K}}_{moir\overset{´}{e}}^{1}$ and ${\vec{K}}_{moir\overset{´}{e}}^{2}$ vectors rotated by a multiple of 60° with respect to the indicated pair, and would have obtained the same six solutions.

(4) *Then evaluate the moiré vector* ${\vec{L}}_{\mathrm{moir}\overset{´}{e}}=\left(m,n\right)$ *with respect to the lattice vectors of the substrate for each of the six rotated vectors* ${\vec{L}}_{moir\overset{´}{e}}=\left(r,s\right)$ *obtained in step (3). This is done by means of* Table 1 *according to the respective augmentation factor. Since the transformations in* Table 1 *are no rotations these six pairs of vectors are not symmetrically equivalent. They belong to six different moiré structures that are mathematically valid solutions of the system of equations above*.

In the example, the augmentation factor was 3, so that we obtain, using $\left(m,n\right)=\left(r-2,s-1\right)$ from Table 1, ${\vec{L}}_{\mathrm{moir}\overset{´}{e}}=\left(m,n\right)$ =(4,1), (2,5), (−4,3), (−8,−3), (−6,−7), (0,−5).

(5) *Calculate the lattice parameters of the six obtained moiré structures. Useful values are the length of* ${\vec{L}}_{\mathrm{moir}\overset{´}{e}}$ *in units of the lattice constant of the substrate [eq. (25)] and of the adsorbate [eq. (26)], the ratio of the lattice constants of the substrate and the adsorbate* x *[eq. (27)], and the coverage* Θ *[eq. (28)]. Further useful values are the rotational angle* $\Phi_{\mathrm{sub}}$ *of the moiré lattice with respect to the substrate lattice [eq. (29], the rotational angle* $\Phi_{\mathrm{ad}}$ *of the moiré lattice with respect to the lattice of the adsorbed layer [eq. (30)], and* φ *, the rotational angle of the adsorbed layer with respect to the substrate [eq. (31)]*.

Table 3 shows the results for the present example. One can see that the six solutions, which are all mathematically valid, vary widely in their lattice parameters. (This is except for $L_{\mathrm{moir}\overset{´}{e}}/a_{\mathrm{ad}}$ , which is the same for all six solutions, and for the $\Phi_{\mathrm{ad}}$ values, which only differ by multiples of 60°. These exceptions result from the fact that these lattice parameters only contain the adsorbed layer to which nothing else than rotational operations were applied.)

**Table 3:** Lattice parameters of the six solutions for the moiré structure formed by CO on Co(0001) at *p*(CO)=800 mbar and *T*=300 K.

	(m,n)	(r,s)	$L_{\mathrm{moir}\overset{´}{e}}/a_{\mathrm{sub}}$	$L_{\mathrm{moir}\overset{´}{e}}/a_{\mathrm{ad}}$	x	Θ	$\Phi_{\mathrm{sub}}$	$\Phi_{\mathrm{ad}}$	φ
1	(4,1)	(6,2)	$\sqrt{13}$	$\sqrt{28}$	1.468	2.15	+13.9°	+19.1°	−5.2°
2	(2,5)	(4,6)	$\sqrt{19}$	$\sqrt{28}$	1.214	1.47	+96.6°	+79.1°	+17.5°
3	(−4,3)	(−2,4)	$\sqrt{37}$	$\sqrt{28}$	0.870	0.76	+154.7°	+139.1°	+15.6°
4	(−8,−3)	(−6,−2)	$\sqrt{49}$	$\sqrt{28}$	0.756	0.57	−158.2°	−160.9°	+2.7°
5	(−6,−7)	(−4,−6)	$\sqrt{43}$	$\sqrt{28}$	0.807	0.65	−112.4°	−100.9°	−11.5°
6	(0,−5)	(2,−4)	$\sqrt{25}$	$\sqrt{28}$	1.058	1.12	−60.0°	−40.9°	−19.1°

(6) *To decide which of the six mathematical solutions describes the physical system obtain information about the orientation and the lattice constant of the substrate. As the substrate lattice is mostly not directly visible in the same STM image, this information has to come from separate experiments. Because of the characteristics of STM data this step may be connected with considerable uncertainties. For example, for some STM designs such as the beetle‐type scanner, the orientation of the STM frames with respect to the lattice directions of the sample may vary from experiment to experiment. However, even with considerable error ranges the physically correct moiré structure can often be uniquely identified*.

**Figure 3:**
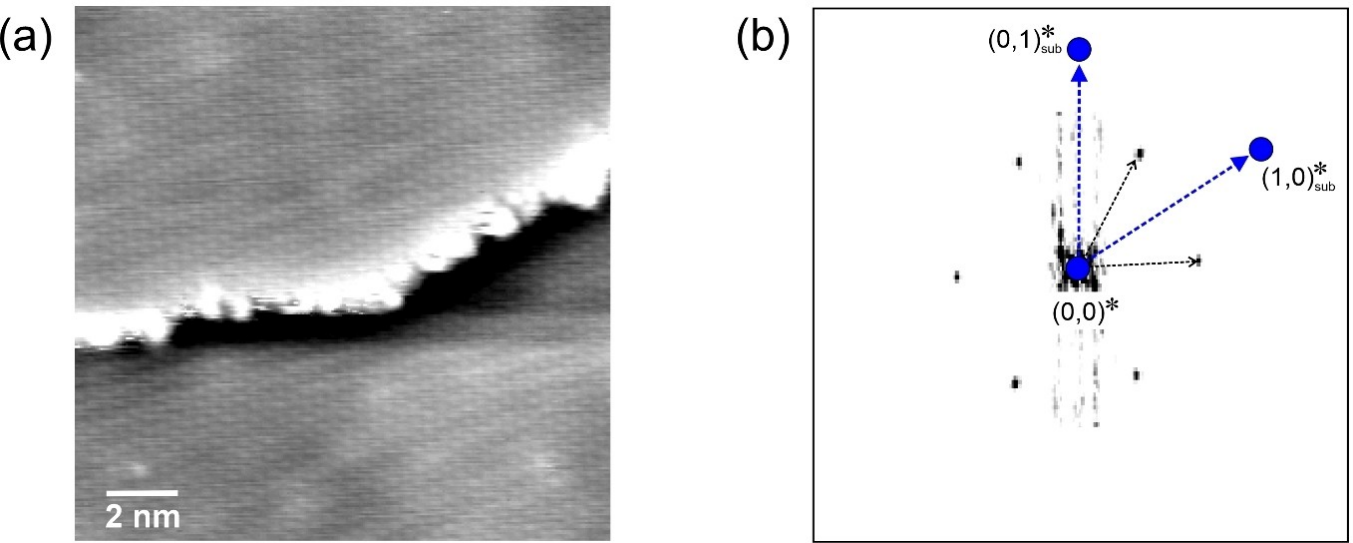
STM experiment on Co(0001) at 1×10^−8^ mbar CO. (a) STM image, showing the hexagonal pattern of the $\left(\sqrt{3}\;x\;\sqrt{3}\right)R30^{\circ }$ structure of adsorbed CO molecules. *T*=300 K, *V_t_*=−1.0 V, *I_t_*=0.7 nA, 150 Å×150 Å. (b) Fourier transform of (a) showing the spots from the $\left(\sqrt{3}\;x\;\sqrt{3}\right)R30^{\circ }$ structure and the corresponding reciprocal lattice vectors. The blue vectors are the reciprocal basis vectors ${\left(1,0\right){}^{*}}_{sub}$ and ${\left(0,1\right){}^{*}}_{sub}$ of the substrate constructed from the reciprocal lattice vectors of the $\left(\sqrt{3}\;x\;\sqrt{3}\right)R30^{\circ }$ structure. Adapted with permission from B. Böller, P. Zeller, S. Günther, J. Wintterlin, *ACS Catal*. **2020**, *10*, 12156–12166 (Ref. [9]). Copyright (2020) American Chemical Society.

In the CO‐on‐Co(0001) study, the substrate orientation could approximately be determined from experiments at lower CO pressures where simple (non‐moiré) structures were observed.^[9]^ Figure 3a shows an STM image recorded at 10^−8^ mbar of CO. The hexagonal pattern is the well known $\left(\sqrt{3}\;x\;\sqrt{3}\right)R30^{\circ }$ structure of CO; Figure 3b is the corresponding Fourier transform. It shows six sharp spots from the $\left(\sqrt{3}\;x\;\sqrt{3}\right)R30^{\circ }$ structure that define the reciprocal lattice vectors of the overlayer. Combinations of these two vectors give the reciprocal basis vectors ${\left(1,0\right){}^{*}}_{sub}$ and ${\left(0,1\right){}^{*}}_{sub}$ of the cobalt substrate. ${\left(1,0\right){}^{*}}_{sub}$ is then transferred to Figure 2d (blue), and the error margins of the length and rotational angle with respect to the vectors from the adsorbed layer are marked (green area). For the length we estimate an error margin of ± 7 %, and for the angle of ± 7°. The relatively high error in φ is caused by the use of a beetle‐type STM in this study, by which the usual errors in determining angles in STM data are enhanced by the varying positions of the scanner with respect to the sample.

However, when one inspects Table 3 one can immediately sort out five of the six solutions. Despite the error margins, it is clear from Figure 2d that ${\left(1,0\right){}^{*}}_{ad}$ is shorter than ${\left(1,0\right){}^{*}}_{sub}$ , so that x<1 . Solutions 1, 2, and 6 can therefore be sorted out because they all have x>1 . It is also clear from Figure 2d that φ is negative from which solutions 3 and 4, which haveφ>0 , can be sorted out. Solution 5, with (m,n) =(−6,−7), is thus identified as the correct moiré structure, a result that would tolerate even higher errors in the lattice constant and orientation of the substrate lattice. If the substrate lattice were completely unknown, one could at least sort out solutions 1, 2, and 6 because they all have Θ>1 which is unphysical for adsorbed CO on Co(0001). (Instead of using the equations from Table 1 one can also solve the structure by a graphical analysis of Figure 2d; see Supporting Information.)

(7) *Finally, to label the moiré structure by the matrix notation and to draw a model, take the vector* ${\vec{L}}_{\mathrm{moir}\overset{´}{e}}=\left(m,n\right)$ *identified as the correct solution in step (6) and rotate it five times by multiples of 60°, using the* $\left(m,n\right)$ *column in* Table 2 *. Of the six obtained vectors, any tuple of vectors with an included angle of 120° provides a valid set of matrix elements* m *and* n *for the matrix* 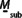 *. To label the structure by the matrix* 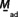 *rotate the vector* ${\vec{L}}_{\mathrm{moir}\overset{´}{e}}=\left(r,s\right)$ *concurrently with* ${\vec{L}}_{\mathrm{moir}\overset{´}{e}}=\left(m,n\right)$ *to obtain the matrix elements* r *and* s *. The parameters required for Wood's nomenclature are already contained in the set of values generated in step (5), namely* $L_{\mathrm{moir}\overset{´}{e}}/a_{\mathrm{sub}}$ *and* $\Phi_{\mathrm{sub}}$ .

For the example treated here, the results of the rotations of ${\vec{L}}_{moir\overset{´}{e}}=\left(m,n\right)$ =(−6,−7) are given in Table 4. To label the structure with the matrix notation, one could, e. g., set up the substrate matrix 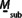 with the matrix elements taken from the 0°/120° vector tuple(35)
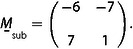



**Table 4:** Moiré lattice vectors, obtained by rotating the vectors from solution 5 in Table 3, ${\vec{L}}_{moir\overset{´}{e}}$ =$\left(m,n\right)$ =(−6,−7) and ${\vec{L}}_{moir\overset{´}{e}}$ =$\left(r,s\right)$ =(−4,−6), by multiples of 60°, using the construction rules of Table 2.

Rotation angle	${\vec{L}}_{moir\overset{´}{e}}=\left(m,n\right)$	${\vec{L}}_{moir\overset{´}{e}}=\left(r,s\right)$
0°	(−6,−7)	(−4,−6)
60°	(1,−6)	(2,−4)
120°	(7,1)	(6,2)
180°	(6,7)	(4,6)
240°	(−1,6)	(−2,4)
300°	(−7,−1)	(−6,−2)

For the construction of the corresponding adsorbate layer matrix 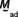 it has to be observed that one has to use the vector tuple with the same rotation angles, i. e.,(36)
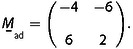



Any other tuple of vectors from Table 4 with included angles of 120° also gives valid 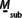 and 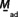 matrices. [Two asides: Firstly, the relation $\left(m,n\right)=\left(r-2,s-1\right)$ (Table 1), which we have used for the analysis of the current case of a tripled cell, only holds for the 0°‐rotated pair of vectors ${\vec{L}}_{moir\overset{´}{e}}=\left(m,n\right)$ and ${\vec{L}}_{moir\overset{´}{e}}=\left(r,s\right)$ (Table 4). For the rotated, other pairs of vectors the relations between m and n on the one side and r and s on the other change. In general form, these relations can be obtained by inserting the equations from Table 1 into Table 2. For example, for a rotation by 60° and a tripled structure, one obtains $m^{'}=r^{'}-1$ and $n^{'}=s^{'}-2$ . The resulting structures are all identical. Secondly, one could take into account that the Co(0001) substrate as an hcp(0001) surface has only threefold rather than sixfold rotationally symmetry when the bulk is included; the same holds for the (111) surfaces of fcc metals. The six obtained solutions (Table 4) are then no longer equivalent but split into two groups with different energies.]

To label the structure by Wood's nomenclature one uses the entries $L_{\mathrm{moir}\overset{´}{e}}/a_{\mathrm{sub}}=\sqrt{43}$ and $\Phi_{\mathrm{sub}}$ =−112.4° for the identified solution 5 from Table 3 (actually, one uses the angle $\Phi_{\mathrm{sub}}$ with respect to the closest multiple of 60°, in this case −120°). The result is a $\left(\sqrt{43}\;x\;\sqrt{43}\right)R7.6^{\circ }$ structure. A structure model is indicated in Figure 2e.

The lattice parameters finally obtained are the same as those obtained by means of the commensurability plot method in ref.^[9]^ However, what is achieved by the method presented here is to solve the structure just by means a calculator (to evaluate the lattice parameters in Table 3).

#### 2.2.2 Moiré Structure of CO Molecules on Co(0001) Formed at 100 mbar (Example 2)

**Figure 4:**
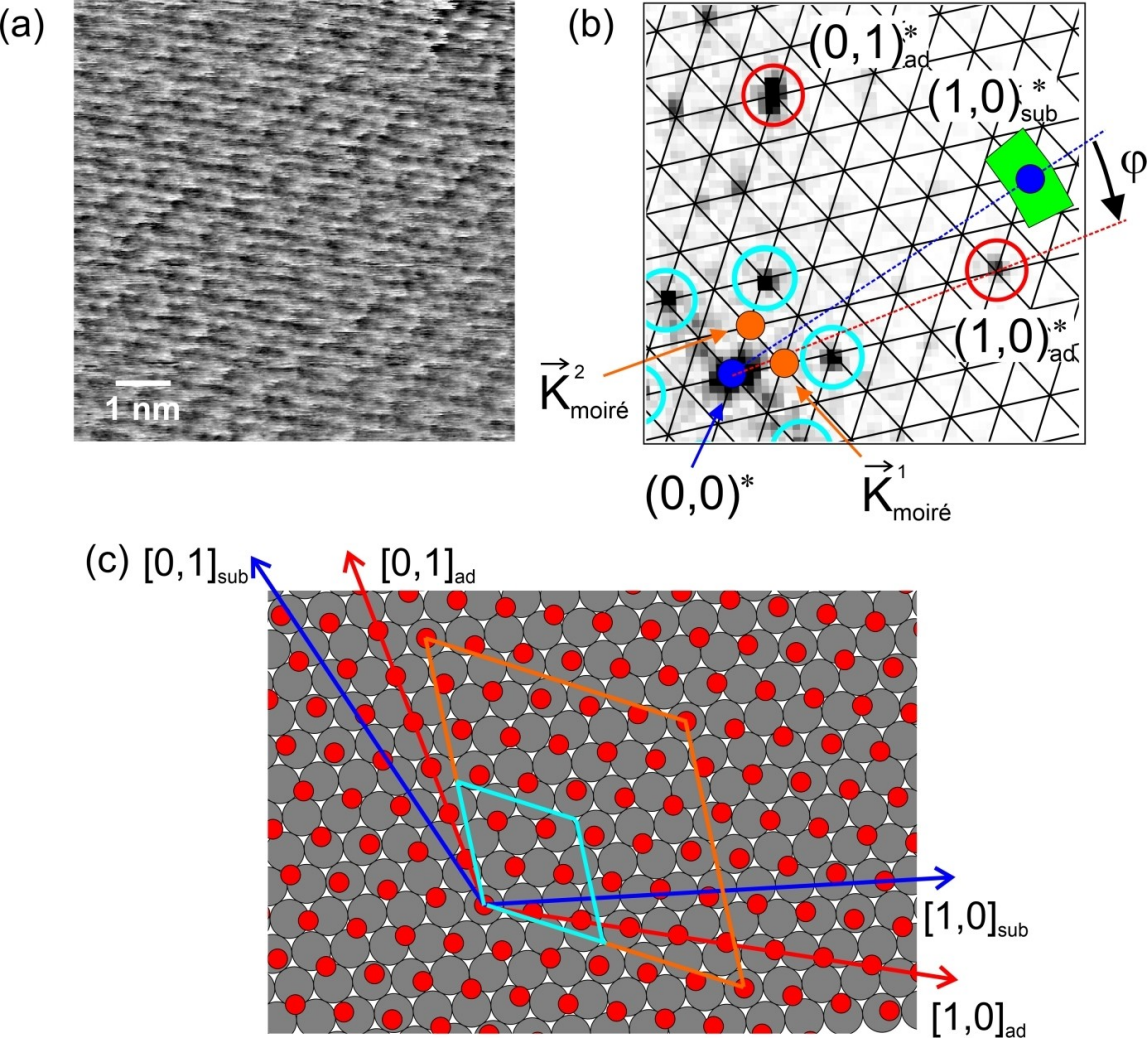
Analysis of a CO moiré structure on Co(0001) at 100 mbar CO. a) STM image of the moiré structure. *T*=300 K, *V_t_*=+1.5 V, *I_t_*=0.7 nA, 80 Å×80 Å. b) One quadrant of the Fourier transform with a four times smaller grid than from the simple cell. All vectors are labelled equivalently to Figure 2d. c) Model of the $\left(7\;x\;7\right)R\bar{21.8^{\circ }}\;$ structure. Color code is the same as in Figure 2e. (Differently from Ref. [9], the Fourier transform has been corrected for some small drift such that the symmetry is exactly hexagonal.) Adapted with permission from B. Böller, P. Zeller, S. Günther, J. Wintterlin, *ACS Catal*. **2020**, *10*, 12156–12166 (Ref. [9]). Copyright (2020) American Chemical Society.

As a second example we investigate a moiré structure from an STM image recorded at a CO pressure of 100 mbar (Figure 4a). The noise level in this experiment is higher, not an unusual problem in *in situ* STM experiments where the high gas pressure often affects the stability of the tunneling tip. In the SI of Ref. [9] the Fourier transform of the image has already been analyzed in detail, so that we can start here with the results of this part (which corresponds to the first three steps of the recipe).

It was shown that the grid from a single cell again does not reproduce the spots from the CO lattice [step (1)]. A fourfold increase was necessary [step (2)]. The Fourier transform does not show any frequencies from this larger periodicity, in contrast to the first example where weak intermediate spots at higher‐order $\sqrt{3}$ positions already indicate that the actual unit cell is three times larger than suggested by the main moiré spots (Figure 2b). However, we point out that the augmentation step does not rely on the existence of such intermediate spots from the moiré structure but is based on spot positions, in particular of those from the atomic fine structure, with respect to the grid. Like in the first example, the reciprocal moiré lattice vectors defined by the grid led to a matrix in reciprocal space [step (3)]. By inversion and transposing, the real space matrix(37)
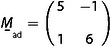



was obtained.^[9]^ The first line of 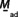 is one of the solutions for ${\vec{L}}_{moir\overset{´}{e}}=\left(r,s\right)$ , expressed in units of the lattice vectors of the adsorbed layer, i. e., (r,s) =(5,−1). ${\vec{L}}_{moir\overset{´}{e}}=\left(r,s\right)$ is then rotated by 60°, 120°, etc. by means of Table 2, giving six solutions, (r,s) =(5,−1), (6,5), (1,6), (−5,1), (−6,−5), (−1,−6).

We briefly work through the next steps of the recipe that have not been applied in Ref. [9]. In step (4), from each of the six vectors ${\vec{L}}_{moir\overset{´}{e}}=\left(r,s\right)$ a vector ${\vec{L}}_{moir\overset{´}{e}}=\left(m,n\right)$ is generated by the transformation $\left(m,n\right)=\left(r-2,s\right)$ (the equation from Table 1 for the quadrupled cell), giving (m,n) =(3,−1), (4,5), (−1,6), (−7.1), (−8,–5), (−3,−6). With m , n , r , and s determined, step (5) is applied to evaluate the full set of lattice parameters for the six solutions. The results are listed in Table 5.

**Table 5:** Lattice parameters of the six solutions for the moiré structure formed by CO on Co(0001) at *p*(CO)=100 mbar and *T*=300 K.

	(m,n)	(r,s)	$L_{\mathrm{moir}\overset{´}{e}}/a_{\mathrm{sub}}$	$L_{\mathrm{moir}\overset{´}{e}}/a_{\mathrm{ad}}$	x	Θ	$\Phi_{\mathrm{sub}}$	$\Phi_{\mathrm{ad}}$	φ
1	(3,−1)	(5,−1)	$\sqrt{13}$	$\sqrt{31}$	1.544	2.38	−13.9°	−8.9°	−5.0°
2	(4,5)	(6,5)	$\sqrt{21}$	$\sqrt{31}$	1.215	1.48	+70.9°	+51.1°	+19.8°
3	(−1,6)	(1,6)	$\sqrt{43}$	$\sqrt{31}$	0.849	0.72	+127.6°	+111.1°	+16.5°
4	(−7,1)	(−5,1)	$\sqrt{57}$	$\sqrt{31}$	0.737	0.54	+173.4°	+171.1°	+2.4°
5	(−8,−5)	(−6,−5)	$\sqrt{49}$	$\sqrt{31}$	0.795	0.63	−141.8°	−128.9°	−12.8°
6	(−3,−6)	(−1,−6)	$\sqrt{27}$	$\sqrt{31}$	1.072	1.15	−90.0°	−68.9°	−21.1°

To decide which of these six solutions describes the present system [step (6)], we again add the reciprocal substrate vector ${\left(1,0\right){}^{*}}_{sub}$ that was determined in Figure 3b to Figure 4b. From the rough length and orientation of ${\left(1,0\right){}^{*}}_{sub}$ one reads out x<1 and a negative φ . Solutions 1, 2, and 6 can therefore be sorted out because they all have x>1 , and solutions 3 and 4 can be sorted out because both have φ>0 . Solution 5 is thus identified as the correct structure. The evaluated coverage of Θ =0.63 is consistent with the fact that in the first example, where the CO pressure is higher, a higher coverage, Θ =0.65, is evaluated.

For the labelling step (7) ${\vec{L}}_{moir\overset{´}{e}}=\left(m,n\right)$ is rotated by the equations in Table 2 to give (m,n) =(−8,−5), (−3,−8), (5,−3), (8,5), (3,8), (−5,3). Then, from these six vectors an arbitrary tuple of 120° rotated vectors is chosen to construct 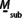 for the matrix notation, for example(38)
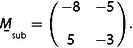



The corresponding adsorbate matrix 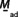 is(39)
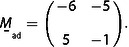



In Wood's nomenclature the structure is labelled using the entries for $L_{\mathrm{moir}\overset{´}{e}}/a_{\mathrm{sub}}=\sqrt{49}$ and *Φ*_sub_=−141.8°. The result is a $\left(7\;x\;7\right)R\bar{21.8^{\circ }}\;$ structure. A structure model is shown in Figure 4c.

Again, the structure is the same as the one previously obtained by the commensurability plot method.^[9]^ The example shows that the recipe leads to a unique result despite the enhanced noise level in the STM image and despite the fact that the Fourier transform does not show the spots from the 4 times larger unit cell.

#### 2.2.3 Moiré Structure of CO Molecules on Co(0001) Formed in a 2 : 1 H_2_/CO Mixture at 950 mbar (Example 3)

**Figure 5:**
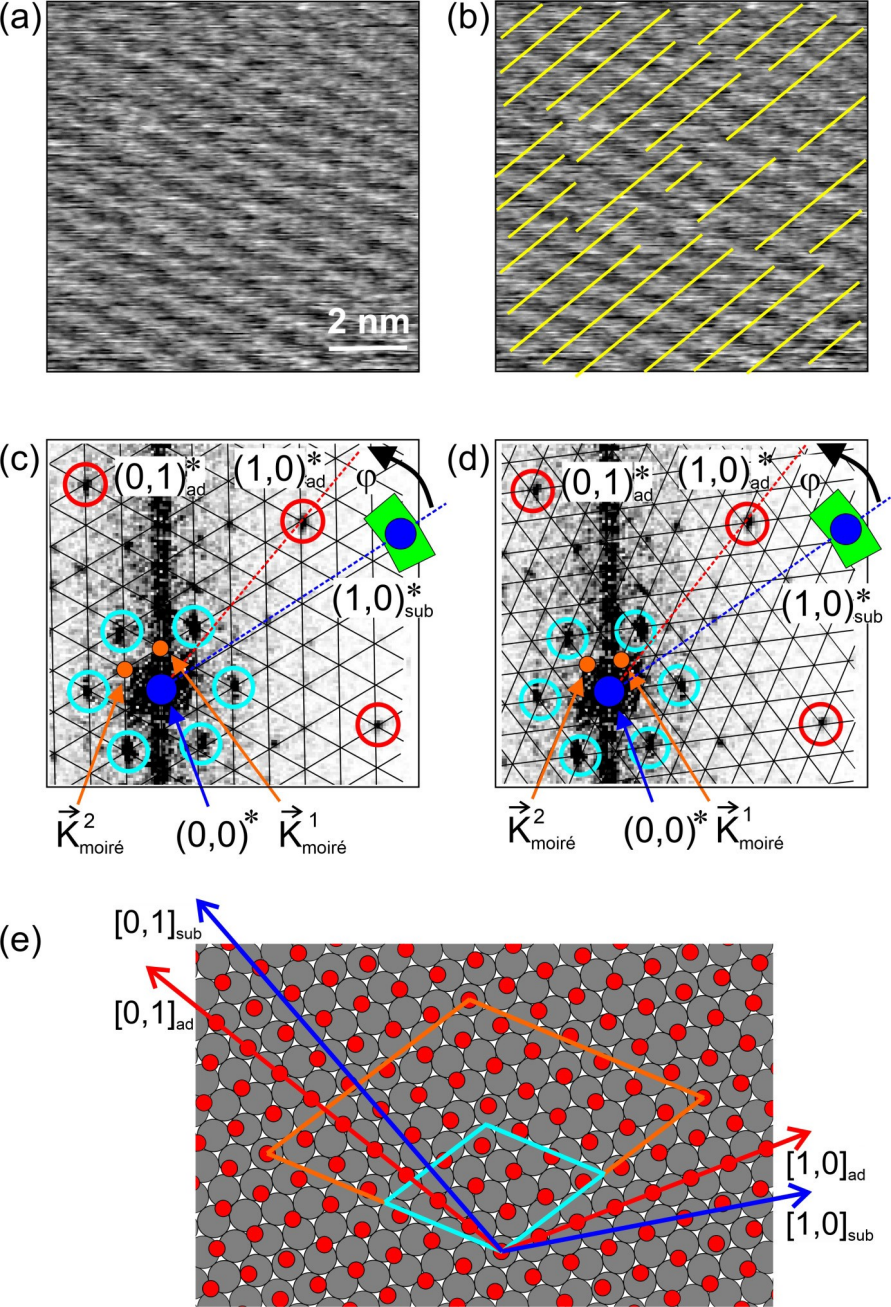
Analysis of a CO moiré structure on Co(0001) in a 2 : 1 H_2_/CO mixture at 950 mbar. a) STM image of the moiré structure. *T*=300 K, *V_t_*=+0.3 V, *I_t_*=0.7 nA, 95 Å×95 Å. The apparent stripe pattern from the upper left to the bottom right is caused by an asymmetric tip. b) Same STM image, with yellow lines indicating ordered rows of moiré unit cells. c) Fourier transform of (a) with superimposed grid from a tripled moiré unit cell. d) Same Fourier transform with superimposed grid from a quadrupled moiré unit cell. e) Model of the $\left(\sqrt{61}\;x\;\sqrt{61}\right)R26.3^{\circ }$ structure. Color code like in Figure 2e. (Differently from Ref. [9], the Fourier transform has been corrected for some small drift such that the symmetry is exactly hexagonal.) Adapted with permission from B. Böller, P. Zeller, S. Günther, J. Wintterlin, *ACS Catal*. **2020**, *10*, 12156–12166 (Ref. [9]). Copyright (2020) American Chemical Society.

The experiment was part of a study of the cobalt‐catalyzed Fischer‐Tropsch synthesis and was therefore performed with a mixture of H_2_ and CO.^[9]^ However, at the temperature of 300 K in this experiment, only CO is adsorbed on the Co(0001) surface – the adsorption of hydrogen is blocked –, so that the moiré structure in Figure 5a is exclusively formed by CO molecules. The signal is somewhat noisy but, as we have seen above, this does not necessarily cause problems. However, in this case, the moiré structure additionally displays some disorder. This is illustrated by the yellow lines in Figure 5b marking ordered rows of moiré unit cells. One can see that at many positions the lines are interrupted and displaced with respect to each other, indicating dislocations in the moiré structure.

In the analysis of the Fourier transform of this image, one finds that the positions of the spots cannot be described by a grid from a simple cell, like in the first two examples [step (1)]. Hence, the cell augmentation method has to be applied [step (2)]. It gives an acceptable result for a grid from a three times larger cell (Figure 5c), but in this case the grid from a four times larger cell describes the positions of most spots not much worse (Figure 5d). It would not be justified excluding this second possibility on the basis of these data. This ambiguity probably has to do with the dislocations seen in the real‐space data that, in reciprocal space, lead to a less well‐defined spot pattern in the Fourier transformation.

The subsequent analysis of the two possible grids that represent two different reciprocal moiré lattices is as above. Because no new aspects are introduced in this part we work through the remaining steps (3) to (7) in the Supporting Information. The critical step (6), in which one has to decide about the physically correct structure, does not cause further ambiguities in both cases, and two clear solutions for moiré structures are obtained. The solution for the three times larger cell is a $\left(\sqrt{43}\;x\;\sqrt{43}\right)R\bar{7.6^{\circ }}$ structure. The solution for the four times larger cell is a $\left(\sqrt{61}\;x\;\sqrt{61}\right)R26.3^{\circ }$ structure. Figure 5e shows a structure model of the latter. Both structures are the same as those obtained by the commensurability plot method.^[9]^

Finding conclusive arguments for or against one of the two structures is difficult. The first solution, the $\left(\sqrt{43}\;x\;\sqrt{43}\right)R\bar{7.6^{\circ }}\;$ structure, is the same structure determined in the 800 mbar experiment, but with a positive rotational angle of the CO layer (φ ) instead of a negative one. As one obtains the $\left(\sqrt{43}\;x\;\sqrt{43}\right)R7.6^{\circ }$ structure by reflecting the $\left(\sqrt{43}\;x\;\sqrt{43}\right)R\bar{7.6^{\circ }}$ structures at the $\sqrt{3}$ direction of the Co(0001) surface, the direction of a mirror plane of the Co(0001) substrate, the two rotated structures are energetically identical. This is consistent with the fact that larger‐scale STM images of other CO moiré structures on Co(0001) often show domains with positive and negative rotations.^[9]^ The observation of a mirror‐image structure at a similar (partial) pressure could therefore be expected and may speak for a $\left(\sqrt{43}\;x\;\sqrt{43}\right)R\bar{7.6^{\circ }}\;$ structure. On the other hand, what may speak for the $\left(\sqrt{61}\;x\;\sqrt{61}\right)R26.3^{\circ }$ structure is that the calculated CO coverage of this structure, Θ =0.64, is exactly between the coverages of Θ =0.63 and 0.65 observed at the lower (100 mbar) and higher CO pressure (800 mbar), respectively, in the two above examples.

We are presenting this example as it points to a limit of the method when the moiré structure lacks long‐range order.

#### 2.2.4 Moiré Structure of a Graphene Layer on Ir(111) (Example 4)

**Figure 6:**
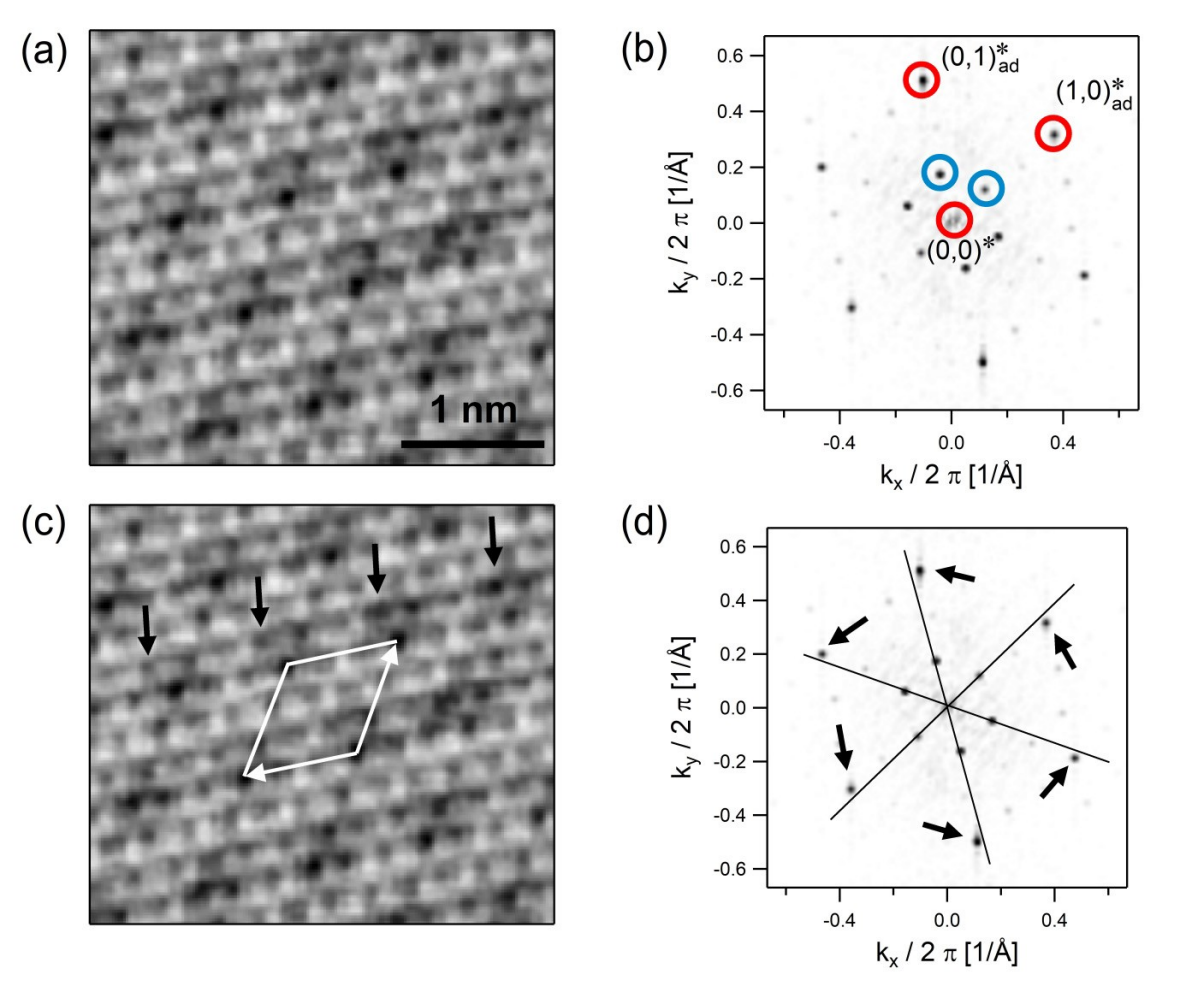
Analysis of an STM image of a graphene monolayer on Ir(111). a) STM image of the moiré structure. *V_t_*=+0.25 V, *I_t_*=30 nA, 30 Å×25 Å. b) Fourier transform of (a). The main spots from the moiré structure are marked by blue circles, and the spots from the graphene lattice are marked by red circles. c) STM image with marked $\left(3\;x\;3\right)$ “motif” of the incommensurate structure (white). Black arrows mark positions that in a real 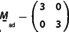 structure would be identical. d) Fourier transform overlaid by the grid constructed on the innermost moiré spots. Arrows indicate the displacements of the spots of the graphene layer from this grid. In this case the Fourier transform has not been corrected for drift. Adapted from P. Zeller, X. Ma, S. Günther, *New. J. Phys*. **2017**, *19*, 013015 (Ref. [39]).

In this example one of the structures formed by graphene monolayers on Ir(111) is investigated.[^18^, ^39^] Figure 6a shows the STM image of a well‐ordered moiré structure, and Figure 6b shows the Fourier transform with marked spots from the moiré lattice (blue circles) and from the fine structure of carbon atoms (red circles).

In Figure 6d the grid constructed from the positions of the moiré spots is superimposed on the Fourier transform (for clarity, only the main axes of the grid are shown). At first sight it appears that in this case step (1) already gives the reciprocal lattice of the moiré structure, i. e., a moiré structure formed by simple cells. It also appears that the moiré structure is aligned to the atomic lattice of the graphene. The further analysis would then be particularly simple and could directly be done by inspection of Figure 6d. From the threefold positions of the spots from the atomic structure with respect to the moiré spots one could at once write down the matrix of the adsorbed layer(40)
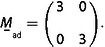



However, this conclusion would be incorrect. Close inspection of Figure 6d shows that the spots from the atomic fine structure are not exactly on the grid but slightly displaced. Moreover, these displacements are not random – as one would expect from experimental errors – but, as shown by the arrows, systematic in the same rotational direction. This rotation is obviously real, meaning that the graphene layer is rotated by a small angle with respect to the moiré lattice. The moiré structure is therefore not formed by simple cells. Applying the cell augmentation method in such a case, where displacements of spots from the grid are very small but not caused by experimental errors, would no longer make sense. The resulting cell would be exceedingly large (larger than the STM image), and the structure is more reasonably classified as incommensurate.

Actually, one can see the incommensurability also in the real‐space STM image (Figure 6c). The black arrows mark cells that in a true 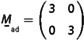 structure would be equivalent. One can see that the cells are, in fact, not translationally symmetric but that their appearance changes from arrow position to arrow position. On the other hand, an 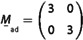 unit cell (white in Figure 6c) is still a good description of the local arrangement of atoms, so that the term “$\left(3\;x\;3\right)$ motif” has been used previously.^[39]^

The example shows that some care has to be taken when the Fourier transform of a moiré structure is analyzed. Small but systematic deviations of spots from a grid may point to an incommensurate structure. The analysis of such structures is beyond of what the method presented here can achieve.

## 3 Conclusions

Commensurate hexagonal moiré structures in STM images can be analyzed by a manual method, without the use of a computer. The method consists of seven steps.

In step (1) a grid is constructed from the first‐order spots of the moiré structure in the Fourier transform of the STM image. If the spots from the atomic fine structure do not fall on the intersection points of this grid, finer grids are constructed in step (2). The finally reached grid defines the reciprocal lattice of the moiré structure. In step (3) a real‐space lattice vector of the moiré structure is constructed, expressed in units of the basis vectors of the adsorbed layer. In step (4) the moiré lattice vector is transformed into a form in which it is expressed in units of the basis vectors of the substrate lattice. This leads to six possible solutions for moiré structures. In step (5) one calculates for each solution the lattice parameters, in particular the ratio of the lattice constant of the substrate and adsorbed layer and the rotational angle of the adsorbed layer with respect to the substrate. When information about the orientation and lattice constant of the substrate is available from reference measurements, a unique solution can be identified [step (6)]. The method automatically provides the parameters required for labelling the moiré structure by the matrix notation and by Wood's nomenclature [step (7)].

In the examples investigated, the results of the method were relatively robust with respect to errors in the orientation of the substrate, and an enhanced noise level did not prevent finding a unique solution. The method reached a limit in an example in which the moiré structure displayed poor long‐range order. Some care was required to exclude an incommensurate structure.

Hexagonal moiré structures are a widespread phenomenon that has been observed for many combinations of adsorption layers and surfaces. The method presented here allows researchers to analyze such structures in STM data without having to perform numerical computations. The method is simple and nevertheless rigorous. It also, in a sense, acts forward, in contrast to trial‐and‐error approaches where one basically draws two hexagonal lattices and then varies the lattice constants and rotational angle until the superimposed pattern matches the structure resolved by STM. Such time‐consuming approaches are avoided. Finally, we point out that the lattice parameters of a moiré structure of a physical system are a direct consequence of the underlying interactions. In turn, when precise lattice parameters have been obtained from an experiment, they can be used to extract improved values of these interactions.

## Conflict of interest

The authors declare no conflict of interest.

## Supporting information

As a service to our authors and readers, this journal provides supporting information supplied by the authors. Such materials are peer reviewed and may be re‐organized for online delivery, but are not copy‐edited or typeset. Technical support issues arising from supporting information (other than missing files) should be addressed to the authors.

Supplementary

## References

[1] D. G. Fedak , N. A. Gjostein , Surf. Sci. 1967, 8, 77–97.

[2] D. G. Castner , G. A. Somorjai , Appl. Surf. Sci. 1980, 6, 29–38.

[3] S. C. Fain Jr. , M. D. Chinn , R. D. Diehl , Phys. Rev. B 1980, 21, 4170–4172.

[4] K. M. Ostyn , C. B. Carter , Surf. Sci. 1982, 121, 360–374.

[5] C. G. Shaw , S. C. Fain Jr. , M. D. Chinn , Phys. Rev. Lett. 1978, 41, 955–957.

[6] K. Kern , R. David , R. L. Palmer , G. Comsa , Phys. Rev. Lett. 1986, 56, 620–623. 10033241 10.1103/PhysRevLett.56.620

[7] H. I. Li , K. J. Franke , J. I. Pascual , L. W. Bruch , R. D. Diehl , Phys. Rev. B 2009, 80, 085415.

[8] S. R. Longwitz , J. Schnadt , E. K. Vestergaard , R. T. Vang , I. Stensgaard , H. Brune , F. Besenbacher , J. Phys. Chem. B 2004, 108, 14497–14502.

[9] B. Böller , P. Zeller , S. Günther , J. Wintterlin , ACS Catal. 2020, 10, 12156–12166.

[10] T. A. Land , T. Michely , R. J. Behm , J. C. Hemminger , G. Comsa , Surf. Sci. 1992, 264, 261–270.

[11] S. Marchini , S. Günther , J. Wintterlin , Phys. Rev. B 2007, 76, 075429.

[12] P. W. Sutter , J.-I. Flege , E. A. Sutter , Nat. Mater. 2008, 7, 406–411. 18391956 10.1038/nmat2166

[13] A. T. N′Diaye , J. Coraux , T. N. Plasa , C. Busse , T. Michely , New J. Phys. 2008, 10, 043033.

[14] S.-Y. Kwon , C. V. Ciobanu , V. Petrova , V. B. Shenoy , J. Bareño , V. Gambin , I. Petrov , S. Kodambaka , Nano Lett. 2009, 9, 3985–3990. 19995079 10.1021/nl902140j

[15] B. Wang , M. Caffio , C. Bromley , H. Früchtl , R. Schaub , ACS Nano. 2010, 4, 5773–5782. 20886811 10.1021/nn101520k

[16] L. Gao , J. R. Guest , N. P. Guisinger , Nano Lett. 2010, 10, 3512–3516. 20677798 10.1021/nl1016706

[17] E. Miniussi , M. Pozzo , A. Baraldi , E. Vesselli , R. R. Zhan , G. Comelli , T. O. Menteş , M. A. Niño , A. Locatelli , S. Lizzit , D. Alfè , Phys. Rev. Lett. 2011, 106, 216101. 21699318 10.1103/PhysRevLett.106.216101

[18] P. Zeller , S. Dänhardt , S. Gsell , M. Schreck , J. Wintterlin , Surf. Sci. 2012, 606, 1475–1480.

[19] Y. Zhang , T. Gao , S. Xie , B. Dai , L. Fu , Y. Gao , Y. Chen , M. Liu , Z. Liu , Nano Res. 2012, 5, 402–411.

[20] L. L. Patera , C. Africh , R. S. Weatherup , R. Blume , S. Bhardwaj , C. Castellarin-Cudia , A. Knop-Gericke , R. Schloegl , G. Comelli , S. Hofmann , C. Cepek , ACS Nano 2013, 7, 7901–7912. 23924234 10.1021/nn402927q

[21] H. Lim , J. Jung , H. J. Yang , Y. Kim , Adv. Mat. Int. 2014, 1, 1300080.

[22] W. Auwärter , Surf. Sci. Rep. 2019, 74, 1–95.

[23] X. Ma , S. Günther , Phys. Chem. Chem. Phys. 2018, 20, 21844–21855. 30014054 10.1039/c8cp03197e

[24] T. Wiederholt , H. Brune , J. Wintterlin , R. J. Behm , G. Ertl , Surf. Sci. 1995, 324, 91–105.

[25] C. Günther , J. Vrijmoeth , R. Q. Hwang , R. J. Behm , Phys. Rev. Lett. 1995, 74, 754–757. 10058839 10.1103/PhysRevLett.74.754

[26] T. M. Bernhardt , B. Kaiser , K. Rademann , Surf. Sci. 1998, 408, 86–94.

[27] J. V. Barth , H. Brune , G. Ertl , R. J. Behm , Phys. Rev. B 1990, 42, 9307–9318. 10.1103/physrevb.42.9307 9995168

[28] A. D. Novaco , J. P. McTague , Phys. Rev. Lett. 1977, 38, 1286–1289.

[29] J. P. McTague , A. D. Novaco , Phys. Rev. B 1979, 19, 5299–5306.

[30] H. Shiba , J. Phys. Soc. Jpn. 1979, 46, 1852–1860.

[31] H. Shiba , J. Phys. Soc. Jpn. 1980, 48, 211–218.

[32] P. Bak , Rep. Prog. Phys. 1982, 45, 587–629.

[33] F. Grey , J. Bohr , Europhys. Lett. 1992, 18, 717–722.

[34] A. Tkatchenko , Phys. Rev. B 2006, 74, 035428.

[35] A. Tkatchenko , Phys. Rev. B 2007, 75, 235411.

[36] K. Hermann , Crystallography and Surface Science: An Introduction for Surface Scientists and Nanoscientists, Wiley-VCH, Weinheim, 2011.

[37] K. Hermann , J. Phys. Condens. Matter 2012, 24, 314210. 22820761 10.1088/0953-8984/24/31/314210

[38] P. Zeller , S. Günther , New J. Phys. 2014, 16, 083028.

[39] P. Zeller , X. Ma , S. Günther , New J. Phys. 2017, 19, 013015.

[40] A. Artaud , L. Magaud , T. Le Quang , V. Guisset , P. David , C. Chapelier , J. Coraux , Sci. Rep. 2016, 6, 25670. 27181495 10.1038/srep25670 PMC4867435

[41] M. Le Ster , T. Märkl , S. A. Brown , 2D Mat. 2020, 7, 011005.

[42] D. Martoccia , P. R. Willmott , T. Brugger , M. Björck , S. Günther , C. M. Schlepütz , A. Cervellino , S. A. Pauli , B. D. Patterson , S. Marchini , J. Wintterlin , W. Moritz , T. Greber , Phys. Rev. Lett. 2008, 101, 126102. 18851393 10.1103/PhysRevLett.101.126102

[43] K. V. Emtsev , F. Speck , T. Seyller , L. Ley , J. D. Riley , Phys. Rev. B 2008, 77, 155303.

