# Supplementary material for: Method for the Manual Analysis of Moiré Structures in STM images
Source: Chemphyschem. 2021 May 4;22(9):870–84. doi: 10.1002/cphc.202001034 (PMC8252427; doi:10.1002/cphc.202001034)
Supplement: Supplementary file 1 — Supplementary [file CPHC-22-870-s001.pdf]

ChemPhysChem

Supporting Information

Method for the Manual Analysis of Moiré Structures in STM images

Sebastian Günther,* Patrick Zeller, Bernhard Böller, and Joost Wintterlin*

Graphical analysis of example 1

One can, instead of using the equations from table 1, also solve a structure graphically. When the reciprocal moiré lattice and the reciprocal basis vector $(1,0)^{*}{}_{\mathrm{sub}}$ of the substrate, together with its error margins, have been obtained, one constructs a triangle in reciprocal space, similarly to the one shown in fig. 1(d) for the case of a simple cell. We demonstrate this by means of example 1, fig. 2(d) of the main text. The triangle consists of the reciprocal basis vectors $(1,0)^{*}{}_{\mathrm{sub}}$ and $(1,0)^{*}{}_{\mathrm{ad}}$ and a displacement vector. Differently from fig. 1(d), the displacement vector for a tripled structure is $\sqrt{3}$ longer than the reciprocal moiré lattice vectors. To draw the triangle, this $\sqrt{3}$ longer vector is applied at the tip of $(1,0)^{*}{}_{\mathrm{ad}}$ in six possible directions that are defined by the lattice points of the reciprocal moiré lattice. Usually the tip of only one of these six vectors will lie in the error margins of the vector $(1,0)^{*}{}_{\mathrm{sub}}$ [the green area in fig. 2(d)]. This is the sought-for displacement vector. The exact length and rotational angle of the vector $(1,0)^{*}{}_{\mathrm{sub}}$ is then also defined. The vector $(0,1)^{*}{}_{\mathrm{sub}}$ is obtained by rotating $(1,0)^{*}{}_{\mathrm{sub}}$ by 60°.

With $(1,0)^{*}{}_{\mathrm{sub}}$ and $(0,1)^{*}{}_{\mathrm{sub}}$ defined on the reciprocal moiré lattice, the following treatment, including the matrix inversion, is equivalent to equations (32) to (34), but applied to the substrate vectors instead of the vectors of the adsorbed layer. It gives the factors m and n, so that, with r and s already determined in step (3), all four factors are known. The analysis of a quadrupled structure is the same except that the displacement vector is 2 times longer than the reciprocal moiré vectors.

The algebraic method described in the main text is usually more straightforward because, in order to sort out the wrong structures, it only requires an approximate knowledge of the rotational angle of the substrate lattice and of the coverage. The latter is usually known from the physics of the system.

Analysis of example 3

For the analysis of the moiré structure resolved at 950 mbar in a H_2/CO mixture we use one quadrant of the Fourier transform [figs. 5(c) and (d) of the main text]. In fig. 5(c) the Fourier transform is superimposed by the grid from the tripled cell, in fig. 5(d) it is superimposed by the grid from the quadrupled cell. For the tripled cell the reciprocal CO vectors extracted from fig. 5(c) are

$$(1,0)^{*}{}_{\mathrm{ad}} = 6\,\vec{K}^{1}_{\mathrm{moiré}} - 4\,\vec{K}^{2}_{\mathrm{moiré}} \quad \text{and} \quad (0,1)^{*}{}_{\mathrm{ad}} = 4\,\vec{K}^{1}_{\mathrm{moiré}} + 2\,\vec{K}^{2}_{\mathrm{moiré}}. \qquad \text{(S1)}$$

Solving for $\vec{K}^{1}_{\mathrm{moiré}}$ and $\vec{K}^{2}_{\mathrm{moiré}}$ gives

$$\vec{K}^{1}_{\mathrm{moiré}} = \frac{1}{14}(1,0)^{*}{}_{\mathrm{ad}} + \frac{2}{14}(0,1)^{*}{}_{\mathrm{ad}} \text{ and } \vec{K}^{2}_{\mathrm{moiré}} = -\frac{2}{14}(1,0)^{*}{}_{\mathrm{ad}} + \frac{3}{14}(0,1)^{*}{}_{\mathrm{ad}}, \qquad \text{(S2)}$$

and the corresponding reciprocal space matrix

$$\underline{M}^{*}_{\mathrm{ad}} = \begin{pmatrix} \frac{1}{14} & \frac{2}{14} \\ -\frac{2}{14} & \frac{3}{14} \end{pmatrix}. \quad \text{(S3)}$$

Transforming $\underline{M}^{*}_{\mathrm{ad}}$ to the real-space matrix $\underline{M}_{\mathrm{ad}}$ gives

$$\underline{M}_{\mathrm{ad}} = \begin{pmatrix} 6 & 4 \\ -4 & 2 \end{pmatrix}. \quad \text{(S4)}$$

The (r, s) vector from the first line of the matrix is rotated six times, giving (r, s) = (6,4), (2,6), (-4,2), (-6,-4), (-2,-6), (4,-2). Using $(m, n) = (r - 2, s - 1)$ for the tripled cell from table 1 (main text) gives (m, n) = (4,3), (0,5), (-6,1), (-8,-5), (-4,-7), (2,-3). The six solutions for m, n, r, and s, are then used to evaluate the lattice parameters $L_{\mathrm{moiré}}/a_{\mathrm{sub}}$, $L_{\mathrm{moiré}}/a_{\mathrm{ad}}$, x, Θ, Φ_{sub}, Φ_{ad}, and φ, which are entered into table S1.

Table S1. Lattice parameters of the six solutions for the moiré structure formed by CO on Co(0001) in a 2:1 mixture of CO and H_2; the total pressure is 950 mbar and the temperature is 300 K. The lattice parameters are evaluated for the tripled structure.

	(m, n)	(r, s)	$\frac{L_{\mathrm{moiré}}}{a_{\mathrm{sub}}}$	$\frac{L_{\mathrm{moiré}}}{a_{\mathrm{ad}}}$	x	Θ	Φ_{sub}	Φ_{ad}	φ
1	(4,3)	(6,4)	$\sqrt{13}$	$\sqrt{28}$	1.468	2.15	+46.1°	+40.9°	+5.2°
2	(0,5)	(2,6)	$\sqrt{25}$	$\sqrt{28}$	1.058	1.12	+120.0°	+100.9°	+19.1°
3	(-6,1)	(-4,2)	$\sqrt{43}$	$\sqrt{28}$	0.807	0.65	+172.4°	+160.9°	+11.5°
4	(-8,-5)	(-6,-4)	$\sqrt{49}$	$\sqrt{28}$	0.756	0.57	-141.8°	-139.1°	-2.7°
5	(-4,-7)	(-2,-6)	$\sqrt{37}$	$\sqrt{28}$	0.870	0.76	-94.7°	-79.1°	-15.6°
6	(2,-3)	(4,-2)	$\sqrt{19}$	$\sqrt{28}$	1.214	1.47	-36.6°	-19.1°	-17.5°

Figure 5(c) also shows the vector $(1{,}0)^{*}{}_{\mathrm{sub}}$ which was transferred fom fig. 3(b) of the main text. From the rough length of this vector x is smaller than one, by which solutions 1, 2, and 6 are sorted out, and from the rough orientation φ is positive, by which solutions 4 and 5 are sorted out. The remaining solution 3 can be labelled in Wood's nomenclature as a $(\sqrt{43}\ x\ \sqrt{43})R\overline{7.6°}$ structure.

The corresponding equations for the quadrupled cell [fig. 5(d)] are

$$(1{,}0)^{*}{}_{\mathrm{ad}} = 7\,\vec{K}^{1}_{\mathrm{moiré}} - 2\,\vec{K}^{2}_{\mathrm{moiré}} \quad \text{and} \quad (0{,}1)^{*}{}_{\mathrm{ad}} = 2\,\vec{K}^{1}_{\mathrm{moiré}} + 5\,\vec{K}^{2}_{\mathrm{moiré}}, \quad \text{(S5)}$$

$$\vec{K}^{1}_{\mathrm{moiré}} = \frac{5}{39}(1{,}0)^{*}{}_{\mathrm{ad}} + \frac{2}{39}(0{,}1)^{*}{}_{\mathrm{ad}} \quad \text{and} \quad \vec{K}^{2}_{\mathrm{moiré}} = -\frac{2}{39}(1{,}0)^{*}{}_{\mathrm{ad}} + \frac{7}{39}(0{,}1)^{*}{}_{\mathrm{ad}}, \quad \text{(S6)}$$

$$\underline{M}_{ad}^* = \begin{pmatrix} \frac{5}{39} & \frac{2}{39} \\ -\frac{2}{39} & \frac{7}{39} \end{pmatrix}, \qquad \text{(S7)}$$

$$\underline{M}_{ad} = \begin{pmatrix} 7 & 2 \\ -2 & 5 \end{pmatrix}. \qquad \text{(S8)}$$

The rotations of (r, s) give (r, s) = (7,2), (5,7), (-2,5), (-7,-2), (-5,-7), (2,-5), and the transformations $(m, n) = (r - 2, s)$ for the quadrupled cell give (m, n) = (5,2), (3,7), (-4,5), (-9,-2), (-7,-7), (0,-5). The resulting lattice parameters are collected in table S2.

Table S2. Lattice parameters of the six solutions for the moiré structure formed by CO on Co(0001) in a 2:1 mixture of H_2 and CO; the total pressure is 950 mbar and the temperature is 300 K. The lattice parameters are evaluated for the quadrupled structure.

	(m, n)	(r, s)	$\frac{L_{moiré}}{a_{sub}}$	$\frac{L_{moiré}}{a_{ad}}$	x	Θ	Φ_{sub}	Φ_{ad}	φ
1	(5,2)	(7,2)	$\sqrt{19}$	$\sqrt{39}$	1.433	2.05	+23.4°	+16.1°	+7.3°
2	(3,7)	(5,7)	$\sqrt{37}$	$\sqrt{39}$	1.027	1.05	+94.7°	+76.1°	+18.6°
3	(-4,5)	(-2,5)	$\sqrt{61}$	$\sqrt{39}$	0.800	0.64	+146.3°	+136.1°	+10.2°
4	(-9,-2)	(-7,-2)	$\sqrt{67}$	$\sqrt{39}$	0.763	0.58	-167.8°	-163.9°	-3.9°
5	(-7,-7)	(-5,-7)	$\sqrt{49}$	$\sqrt{39}$	0.892	0.80	-120.0°	-103.9°	-16.1°
6	(0,-5)	(2,-5)	$\sqrt{25}$	$\sqrt{39}$	1.249	1.56	-60.0°	-43.9°	-16.1°

Experimentally, x is smaller than one, in contrast to solutions 1, 2, and 6, and φ is positive, in contrast to solutions 4 and 5. The remaining solution 3 can be labelled in Wood's nomenclature as a $(\sqrt{61}\, x\, \sqrt{61})R26.3°$ structure.
